# Supplementary material for: Aberrant Signaling Pathways in Sinonasal Intestinal-Type Adenocarcinoma
Source: Cancers (Basel). 2021 Oct 7;13(19):5022. doi: 10.3390/cancers13195022 (PMC8507674; doi:10.3390/cancers13195022)
Supplement: Supplementary file 1 [file cancers-13-05022-s001.zip › cancers-1416120-supplementary.pdf]

Table S1. Somatic and loss of heterozygosity (LOH) variants found in the 29 tumor/germline matched cases.

Case	Subtype	Gene	c.Hgvs	p.Hgvs	Variant Classification	Blood AF	Tumor AF
**1**	Colonic	ERBB3	c.217G>A	p.Asp73Asn	Missense	0	0.32
		LRP1B	c.2152C>A =	p.His718Asn	Missense	0	0.29
		LRP1B	c.10844G>C	p.Gly3615Ala	Missense (LOH)	0.37	0.78
**3**	Solid	ATM	c.1744T>C	p.Phe582Leu	Missense (LOH)	0.44	0.95
		BRCA2	c.9976A>T	p.Lys3326*	Nonsense (LOH)	0.51	0.89
**4**	Mucinous	DNMT3A	c.1895A>G =	p.His632Arg	Missense	0	0.19
**6**	Colonic	KRAS	c.38G>A	p.Gly13Asp	Missense	0	0.53
		APC	c.5826_5829delCAGA	p.Asp1942Glufs*27	Frameshift Deletion	0	0.38
		ATM	c.3161C>G	p.Pro1054Arg	Missense (LOH)	0.41	0.82
**7**	Colonic	KRAS	c.35G>A	p.Gly12Asp	Missense	0	0.43
		CDKN2A	c.172C>T	p.Arg58*	Nonsense	0	0.81
		FOXA1	c.442C>G	p.Leu148Val	Missense (LOH)	0.50	0.85
**8**	Colonic	BRAF	c.1780G>A	p.Asp594Asn	Missense	0	0.31
		PIK3CA	c.1637A>G	p.Gln546Arg	Missense	0	0.29
		CTNNB1	c.1004A>T	p.Lys335Ile	Missense	0	0.59
**10**	Mucinous	NOTCH2	c.4733G>A	p.Arg1578His	Missense	0	0.27
		PDGFRA	c.1364A>G	p.Lys455Arg	Missense	0	0.28
		KIT	c.1039C>A =	p.Gln347Lys	Missense	0	0.23
		PIK3CA	c.331A>G	p.Lys111Glu	Missense	0	0.25
		PIK3CA	c.1361A>G	p.Asp454Gly	Missense	0	0.26
		ATM	c.497-2A>T =		Intronic Splicing	0	0.28
		KMT2A	c.472dupA =	p.Arg158Lysfs*12	Frameshift Insertion	0	0.25
		KMT2A	c.7255G>T =	p.Glu2419*	Nonsense	0	0.26
		ESR1	c.1057G>T =	p.Glu353*	Nonsense	0	0.19
		PIK3R2	c.1010+3A>T =		Intronic Splicing	0	0.24
**11**	Papillary	ERBB4	c.2008A>G	p.Thr670Ala	Missense	0	0.28
		PIK3CA	c.3140A>G	p.His1047Arg	Missense	0	0.30
		SMO	c.2285G>A	p.Arg762His	Missense	0	0.29
		CDKN1B	c.376G>T =	p.Glu126*	Nonsense	0	0.29
		KRAS	c.35G>A	p.Gly12Asp	Missense	0	0.27
**12**	Colonic	ROS1	c.6116G>A	p.Arg2039His	Missense	0	0.22
		NF1	c.6709C>T	p.Arg2237*	Nonsense	0	0.71
		BRCA1	c.4039A>G	p.Arg1347Gly	Missense (LOH)	0.53	0.86
		NOTCH3	c.3399C>A	p.His1133Gln	Missense (LOH)	0.44	0.86
		JAK3	c.2164G>A	p.Val722Ile	Missense (LOH)	0.50	0.86
**13**	Colonic	ERBB2	c.929C>T	p.Ser310Phe	Missense	0	0.10
**15**	Mucinous	NOTCH2	c.4888C>T	p.Arg1630Cys	Missense	0	0.12
**16**	Colonic	FOXL2	c.695C>T	p.Ala232Val	Missense	0	0.30
		APC	c.4585C>T	p.Gln1529*	Nonsense	0	0.40
		MAP2K1	c.622G>C =	p.Asp208His	Missense	0	0.29
		NF1	c.7946C>G	p.Ser2649*	Nonsense	0	0.42
**17**	Mucinous	PIK3CA	c.2176G>A	p.Glu726Lys	Missense	0	0.26
		APC	c.4618G>T	p.Glu1540*	Nonsense	0	0.42
**18**	Papillary	BRCA1	c.4600G>A	p.Val1534Met	Missense (LOH)	0.47	0.76
**21**	Colonic	MTOR	c.6607G>A	p.Gly2203Ser	Missense	0	0.23
		IDH1	c.394C>T	p.Arg132Cys	Missense	0	0.29
		ATM	c.2051A>C =	p.Gln684Pro	Missense	0	0.43
		NF1	c.7267dupA	p.Thr2423Asnfs*4	Frameshift Insertion	0	0.36
**22**	Colonic	BRCA1	c.4807_4823delGACTCTGGGGCTCTGTC =	p.Pro1603Argfs*13	Frameshift Deletion	0	0.56
		AR	c.234_239delGCAGCA	p.Gln79_Gln80del	Inframe Deletion	0	0.11
		ERBB3	c.3529C>A	p.Leu1177Ile	Missense (LOH)	0.44	0.92
**23**	Mucinous	AR	c.234_239delGCAGCA	p.Gln79_Gln80del	Inframe Deletion	0	0.13
		LRP1B	c.1604T>C	p.Val535Ala	Missense	0	0.21
**24**	Mucinous	CTNNB1	c.133_135delTCT	p.Ser45del	Inframe Deletion	0	0.51
		FOXP1	c.1144C>A =	p.Pro382Thr	Missense	0	0.30
		PIK3CA	c.2816A>G	p.Asp939Gly	Missense	0	0.25
**25**	Solid	DDR2	c.1323G>A	p.Met441Ile	Missense (LOH)	0.46	0.76
**26**	Colonic	LRP1B	c.4496C>A	p.Thr1499Lys	Missense	0	0.17

		APC	c.4282G>T	p.Gly1428Termfs*1	Nonsense	0	0.37
		AR	c.234_239delGCAGCA	p.Gln79_Gln80del	Inframe Deletion	0	0.12
**28**	Colonic	APC	c.4393_4394delAG	p.Ser1465Trpfs*3	Frameshift Deletion	0	0.31
		ERBB3	c.1064C>G =	p.Thr355Ser	Missense	0	0.30
**29**	Mucinous	EZH2	c.2193C>T	p.Tyr731Tyr	Missense	0	0.25

Only splicing and coding non-silent mutations with a tumor allele frequency >0.1 were considered. AF: Allele Frequency; c.Hgvs: standard HGVS nomenclature to describe the alteration at the DNA level; p.Hgvs: standard HGVS nomenclature to describe the predicted consequence at the protein level;.=: Variants not described in any of the consulted ICGC, COSMIC and Varsome cancer databases (reviewed 19.05.2020).

Table S2. Predicted somatic variants in the 21 tumor-only cases.

Case	Subtype	Gene	c.Hgvs	p.Hgvs	Variant Classification	Tumor AF	FATHMM-MKL score	SIFT Score
**30**	Colonic	EPHA2	c.2319delC =	p.Thr774Profs*37	Frameshift Deletion	0.27	ND	ND
		ATM	c.146C>G	p.Ser49Cys	Missense	0.48	0.50 Damaging	0.002 Damaging
		LRP1B	c.2005A>G	p.Ile669Val	Missense	0.51	0.4544 Neutral	1 Tolerated
**31**	Colonic	NOTCH1	c.4028C>T	p.Ala1343Val	Missense	0.50	0.9836 Damaging	0.199 Tolerated
		KRAS	c.35G>A	p.Gly12Asp	Missense	0.58	0.9787 Damaging	0.002 Damaging
		AR	c.228_239delGCAGCAGCAGCA	p.Gln77_Gln80del	Inframe Deletion	0.17	ND	ND
**33**	Colonic	APC	c.2413C>T	p.Arg805*	Nonsense	0.63	0.8264 Damaging	ND
		KRAS	c.176C>G	p.Ala59Gly	Missense	0.16	0.993 Damaging	0.003 Damaging
**34**	Colonic	FOXP1	c.1135G>A	p.Ala379Thr	Missense	0.49	0.7647 Damaging	0.089 Tolerated
**35**	Mucinous	NOTCH2	c.7223T>A	p.Leu2408His	Missense	0.24	0.8534 Damaging	0.013 Damaging
		LRP1B	c.12161A>C	p.Glu4054Ala	Missense	0.36	0.9664 Damaging	0.736 Tolerated
		PDGFRA	c.39_44delTCTTCT =	p.Leu14_Leu15del	Inframe Deletion	0.11	ND	ND
		AR	c.303_308dupGCAGCA	p.Gln79_Gln80dup	Inframe Duplication	0.10	ND	ND
**36**	Mucinous	ATM	c.998C>T	p.Ser333Phe	Missense	0.46	0.8806 Damaging	0.008 Damaging
		NF1	c.2686delG =	p.Asp896Ilefs*6	Frameshift Deletion	0.12	ND	ND
		AR	c.303_308dupGCAGCA	p.Gln79_Gln80dup	Inframe Duplication	0.10	ND	ND
**37**	Solid	BRCA1	c.2521C>T	p.Arg841Trp	Missense	0.48	0.03177 Neutral	0.003 Damaging
		ROS1	c.2411C>A	p.Thr804Asn	Missense	0.42	0.97 Damaging	0.013 Damaging
		AR	c.1424C>T	p.Ala475Val	Missense	0.99	0.2074 Neutral	0.023 Damaging
**38**	Colonic	AKT1	c.138C>A	p.Asp46Glu	Missense	0.46	0.9444 Damaging	0.223 Tolerated
		TSC2	c.5383C>T	p.Arg1795Cys	Missense	0.44	0.7739 Damaging	0 Damaging
		BRCA1	c.3331_3334delCAAG	p.Gln1111Asnfs*5	Frameshift Deletion	0.38	ND	ND
		CSF1R	c.895G>A	p.Ala299Thr	Missense	0.49	0.6279 Damaging	0.039 Damaging
		MET	c.504G>T	p.Glu168Asp	Missense	0.47	0.6196 Damaging	0.304 Tolerated
		AR	c.237_239delGCA	p.Gln80del	Inframe Deletion	0.12	ND	ND
**39**	Solid	SMO	c.808G>A	p.Val270Ile	Missense	0.48	0.9063 Damaging	1 Tolerated
		PTCH1	c.3487G>A	p.Gly1163Ser	Missense	0.46	0.9875 Damaging	0.065 Tolerated
**40**	Colonic	ATM	c.2932T>C	p.Ser978Pro	Missense	0.50	0.9876 Damaging	0 Damaging
		JAK3	c.2152G>C	p.Val718Leu	Missense	0.47	0.7959 Damaging	0.035 Damaging
**41**	Colonic	FLT3	c.1774G>A	p.Val592Ile	Missense	0.15	0.2952 Neutral	1 Tolerated
		ERBB2	c.1960A>G	p.Ile654Val	Missense	0.71	0.9359 Damaging	0.161 Tolerated
		BRCA1	c.4039A>G	p.Arg1347Gly	Missense	0.26	0.6499 Damaging	0.179 Tolerated
		APC	c.1779G>A	p.Trp593*	Nonsense	0.39	0.9912 Damaging	ND
		JAK3	c.2164G>A	p.Val722Ile	Missense	0.46	0.2303 Neutral	0.029 Damaging
**42**	Colonic	LRP1B	c.13114A>T	p.Asn4372Tyr	Missense	0.48	0.9433 Damaging	0.008 Damaging
		LRP1B	c.11227G>A	p.Gly3743Ser	Missense	0.51	0.9899 Damaging	0.1 Tolerated
		AR	c.237_239delGCA	p.Gln80del	Inframe Deletion	0.12	ND	ND
**43**	Colonic	KRAS	c.35G>A	p.Gly12Asp	Missense	0.28	0.9787 Damaging	0.002 Damaging
		EPHA2	c.2162G>A	p.Arg721Gln	Missense	0.47	0.9092 Damaging	0.12 Tolerated

		TSC2	c.5378G>A	p.Arg1793Gln	Missense	0.51	0.9696 Damaging	0.008 Damaging
		AR	c.237_239delGCA	p.Gln80del	Inframe Deletion	0.13	ND	ND
**44**	Colonic	TSC2	c.5116C>T	p.Arg1706Cys	Missense	0.53	0.7786 Damaging	0.023 Damaging
		APC	c.1746dupA $^{=}$	p.Ser583Ilefs*19	Frameshift Duplication	0.58	ND	ND
**45**	Colonic	ATM	c.1810C>T	p.Pro604Ser	Missense	0.48	0.9573 Damaging	0.107 Tolerated
		ATM	c.4388T>G	p.Phe1463Cys	Missense	0.48	0.9658 Damaging	0.001 Damaging
		NTRK1	c.16C>T	p.Arg6Trp	Missense	0.66	0.7065 Damaging	0.006 Damaging
		LRP1B	c.12003G>T	p.Trp4001Cys	Missense	0.29	0.9847 Damaging	0.006 Damaging
**46**	Papillary	KMT2A	c.10648G>A	p.Gly3550Arg	Missense	0.48	0.8588 Damaging	0.002 Damaging
		ERBB2	c.1157C>A	p.Ala386Asp	Missense	0.98	0.1996 Neutral	0.162 Tolerated
		ERBB2	c.2033G>A	p.Arg678Gln	Missense	0.98	0.9412 Damaging	0.099 Tolerated
		NTRK1	c.2339G>A	p.Arg780Gln	Missense	0.50	0.2051 Neutral	0.231 Tolerated
		NOTCH3	c.6532C>T	p.Pro2178Ser	Missense	0.49	0.1974 Neutral	0.569 Tolerated
**47**	Colonic	TSC2	c.5383C>T	p.Arg1795Cys	Missense	0,51	0.7739 Damaging	0 Damaging
		BRCA1	c.1367T>C	p.Ile456Thr	Missense	0,49	0.7227 Damaging	0.033 Damaging
**48**	Solid	IL7R	c.760G>A	p.Ala254Thr	Missense	0,48	0.02848 Neutral	0.169 Tolerated
		MTOR	c.985G>A	p.Ala329Thr	Missense	0,46	0.9921 Damaging	0.48 Tolerated
**49**	Solid	BRCA2	c.430G>T	p.Val144Phe	Missense	0,24	0.2751 Neutral	0.008 Damaging
		BRCA2	c.5299A>T $^{=}$	p.Lys1767*	Nonsense	0,41	0.2051 Neutral	ND
		EPHA2	c.334G>A	p.Ala112Thr	Missense	0,52	0.7379 Damaging	0.425 Tolerated

Only splicing and coding non-silent mutations with a tumor allele frequency >0.1 that appear in at least one of the consulted ICGC, COSMIC and Varsomec cancer databases or have a known protein effect were considered. AF: Allele Frequency; c.Hgvs: standard HGVS nomenclature to describe the alteration predicted consequence at the DNA level; p.Hgvs: standard HGVS nomenclature to describe the alteration predicted consequence at the protein level; FATHMM: Functional Analysis through Hidden Markov Models, an in-silico tool that predicts the effects of protein missense mutations; MKL: in addition to FATHMM predicts noncoding effects by integrating functional annotation information from the ENCODE, range 0 to 1; SIFT: sorts intolerant from tolerant, an in-silico prediction tool for nonsynonymous variants based on sequence homology derived from closely related sequences, range 0 to 1 with values less than 0.05 usually considered intolerant. $^{=}$: Variants not described in any of the consulted ICGC, COSMIC and Varsome cancer databases (rev.19.05.2020).

Table S3. Copy number gains in the 29 tumor/germline matched cases.

Case	Subtype	Chromosome	Start	End	Gene	Copy Number	Variant Type	Affected Region
**3**	Solid	11	69641292	69651343	CCND1	4	Gain	Whole Gene
		11	108227564	108365572	ATM	8	Gain	Whole Gene
		7	116695693	116796137	MET	20	Gain	Whole Gene
		8	38413559	38461172	FGFR1	4	Gain	Whole Gene
**4**	Mucinous	8	38413559	38461172	FGFR1	4	Gain	Whole Gene
**10**	Mucinous	4	1793861	1807700	FGFR3	4	Gain	Whole Gene
**12**	Colonic	1	114708474	114716224	NRAS	10	Gain	Whole Gene
		13	28003992	28100572	FLT3	5	Gain	Whole Gene
		8	38413559	38461172	FGFR1	4	Gain	Whole Gene
**17**	Mucinous	6	36677776	36685859	CDKN1A	4	Gain	Whole Gene
		6	117288478	117425714	ROS1	4	Gain	Whole Gene
		6	151807898	152098980	ESR1	4	Gain	Whole Gene
**18**	Papillary	17	39699501	39728058	ERBB2	5	Gain	Whole Gene
		17	43045607	43124164	BRCA1	5	Gain	Whole Gene
**20**	Colonic	11	69641292	69651343	CCND1	4	Gain	Whole Gene
		3	38138640	38141352	MYD88	4	Gain	Whole Gene
		3	41224002	41239357	CTNNB1	6	Gain	Whole Gene
**21**	Colonic	15	90084201	90102452	IDH2	4	Gain	Whole Gene
		15	98649520	98957455	IGF1R	4	Gain	Whole Gene
**22**	Colonic	1	11106537	11259472	MTOR	4	Gain	Whole Gene
		7	81702512	81770035	HGF	4	Gain	Whole Gene
		7	92615080	92833338	CDK6	4	Gain	Whole Gene
		7	116695693	116796137	MET	4	Gain	Whole Gene
		7	129189136	129212465	SMO	4	Gain	Whole Gene
		7	140734514	140924766	BRAF	4	Gain	Whole Gene
		7	148807518	148847359	EZH2	4	Gain	Whole Gene
**24**	Mucinous	3	41224002	41239357	CTNNB1	4	Gain	Whole Gene
**25**	Solid	1	156815761	156881705	NTRK1	4	Gain	Whole Gene
		1	162718996	162780310	DDR2	4	Gain	Whole Gene
		1	206768573	206772496	IL10	4	Gain	Whole Gene
		1	243499680	243843231	AKT3	4	Gain	Whole Gene
		7	81702512	81770035	HGF	5	Gain	Whole Gene
		7	92615080	92833338	CDK6	6	Gain	Whole Gene

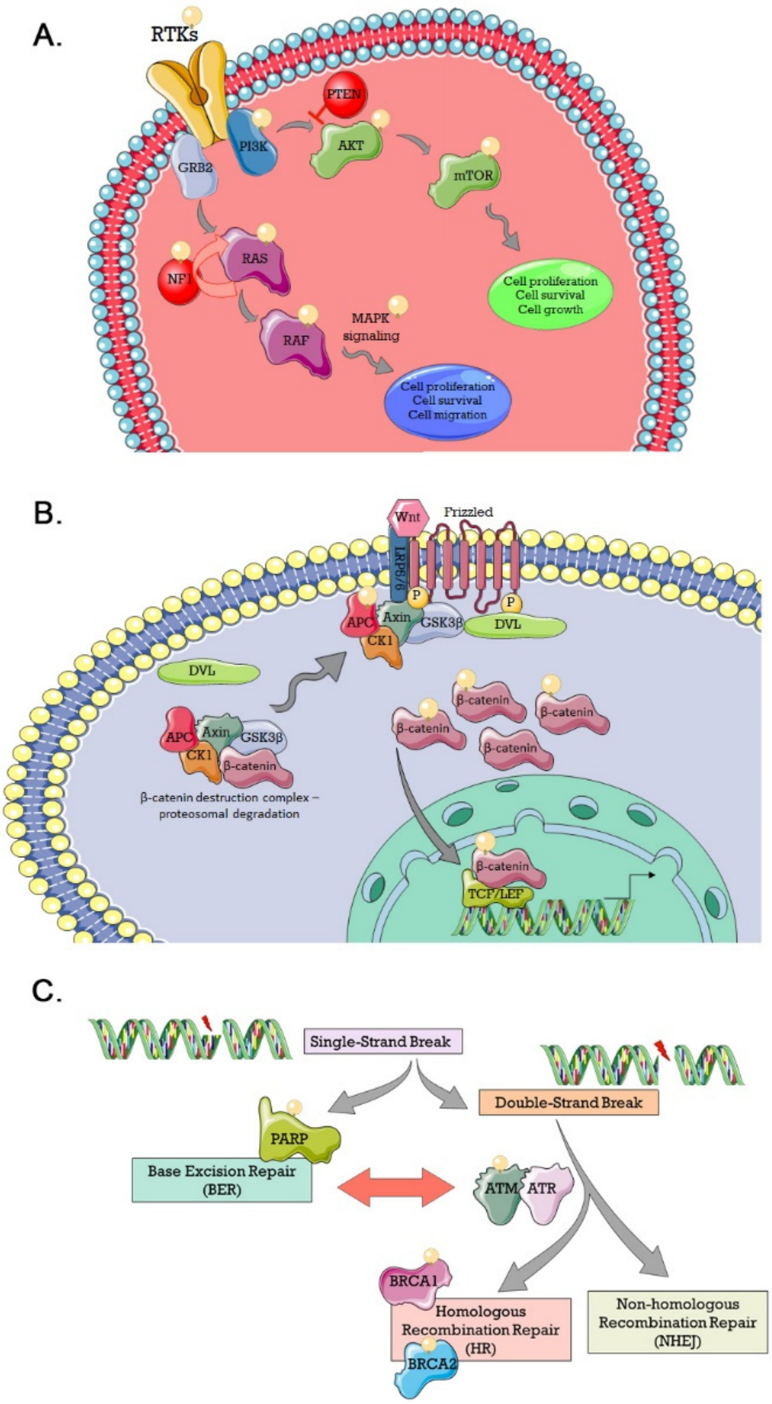

Figure S1. Maps of the signaling pathways discussed in this paper. (A) Combined MAPK and PI3K pathways that can be activated through various RTKs (main manuscript sections 3.4.3, 3.4.4 and 3.4.5); (B) Wnt pathway (main manuscript section 3.4.1); (C) DNA damage response pathway (main manuscript section 3.4.2). The genes/proteins marked by a yellow globe were found mutated in this study.
